# Exploring the role of NOD2 variants in pediatric undifferentiated recurrent fever: a clinical and functional perspective

**DOI:** 10.3389/fimmu.2025.1657782

**Published:** 2025-11-21

**Authors:** Raziye Burcu Taşkın, Arzum Hande Kamiloğlu, Buşra Bara, Gizem Akyol, İlyas Aydın, Gulcin Aytac, Neslihan Edeer Karaca, Güzide Aksu, Afig Berdeli, Vildan Bozok, Necil Kütükçüler

**Affiliations:** 1 Department of Pediatric Rheumatology, Ege University Faculty of Medicine, Izmir, Türkiye; 2 Department of Medical Biology, Ege University Faculty of Medicine, Izmir, Türkiye; 3 Department of Pediatric Immunology, Ege University Faculty of Medicine, Izmir, Türkiye; 4 Molecular Genetics Laboratory, Department of Pediatrics, Ege University Faculty of Medicine, Izmir, Türkiye

**Keywords:** NOD2, autoinflammatory disease, recurrent fever, pediatric, mutation, undifferentiated fever, autoinflammation, cytokines

## Abstract

**Introduction:**

Syndrome of Undifferentiated Recurrent Fever (SURF) is an autoinflammatory disorder with onset in childhood, marked by recurrent episodes of fever without an established molecular diagnosis. Although NOD2 gene variants that are generally considered non-pathogenic are often identified in these patients, their contribution to disease development is still not well understood.

**Methods:**

This study aimed to assess the clinical characteristics, long-term progression, and functional implications of NOD2 variants in a group of twelve children diagnosed with SURF, along with two Blau syndrome cases and two healthy controls. Clinical information was gathered at presentation and during follow-up. Peripheral blood mononuclear cells were examined for cytokine secretion and NF-kB pathway activation, both at baseline and following muramyl dipeptide stimulation, using multiplex cytokine analysis, Western blot, and ELISA.

**Results:**

The median follow-up period was 3.75 years, with most children developing symptoms before 10 years of age. Abdominal pain and limb pain were the most frequent complaints. All patients were treated with colchicine, and selected cases required corticosteroids or disease-modifying antirheumatic drugs. Elevated levels of proinflammatory cytokines, including IL-2, TNF-a, IL- 6, and IL-8, were observed in SURF patients. Our functional studies suggested that variants like R702W, G908R, P268S/V955I, and R702W/P268S might have triggered stronger inflammatory responses, whereas L682F, L1007fs, and R587C might have been linked to diminished cytokine production and lower NF-kB activity. Certain variants, such as A1000T and P268S, appeared to show baseline NF-kB activation with moderate inflammatory activity.

**Discussion:**

Our findings emphasize the clinical and functional diversity of NOD2 variants in SURF and may point to a possible genotype–phenotype relationship that could aid in understanding disease pathways and refining diagnostic approaches.

## 1 Introduction

Systemic autoinflammatory diseases (SAIDs) are defined by dysregulation of the innate immune system and episodes of sterile inflammation (1). Because of overlapping clinical manifestations, diagnosis usually depends on recognizing specific phenotypic patterns, often supported by genetic testing (2). Nevertheless, a notable proportion of individuals presenting with autoinflammatory features do not fulfill the criteria for any established SAID or for conditions such as periodic fever, aphthous stomatitis, pharyngitis, and adenitis (PFAPA), nor do they carry pathogenic mutations in genes associated with hereditary SAIDs. These patients are categorized as having syndrome of undifferentiated recurrent fever (SURF) (3).

This creates particular challenges in interpreting genetic findings in atypical cases with prominent inflammatory features, especially when common variants are identified in pleiotropic genes like *nucleotide-binding oligomerization domain-containing protein 2* (*NOD2*). *NOD2* encodes a cytosolic pattern recognition receptor essential for innate immune defense, as it mediates proinflammatory signaling pathways (4). Mutations in *NOD2* have been associated with granulomatous autoinflammatory conditions, including Blau syndrome (BS), inflammatory bowel disease (IBD), and NOD2-associated autoinflammatory disease (NAID) (4, 5). Recently, non-pathogenic NOD2 variants have been increasingly reported in patients diagnosed with SURF, although the number of cases remains limited (6–9). Across these studies, all patients exhibited recurrent fever, with abdominal pain, rash, and musculoskeletal symptoms as the most frequently reported manifestations (6, 8, 9). However, given the limited evidence to date and the observation that some patients also carried co-existing variants in other genes, the role of non-pathogenic NOD2 variants in shaping clinical features or influencing disease course remains uncertain.

In light of the limited knowledge regarding the inflammatory pathways driving SURF and its diverse clinical presentation, this study aims to characterize the clinical features, treatment outcomes, and functional impact of *NOD2* variants in patients with SURF.

## 2 Materials and methods

### 2.1 Patients and study design

This longitudinal follow-up study, conducted between January 2022 and 2025, included 12 pediatric-onset SURF patients diagnosed in our clinic who harbored non-pathogenic *NOD2* variants. Alongside clinical monitoring, *in vitro* functional studies were performed to assess the effects of these variants on *NOD2*-mediated immune responses.

Patients were classified as having SURF (3) if they exhibited recurrent or persistent fever for at least six months, with or without systemic or organ-specific symptoms, lacked pathogenic mutations associated with hereditary recurrent fevers (HRFs), and did not meet criteria for PFAPA (2), after alternative infectious, neoplastic, and autoimmune conditions were excluded (10). Inclusion criteria also required disease onset before 18 years of age, at least 12 months of follow-up, and detection of a non-pathogenic *NOD2* variant via next-generation sequencing (NGS). All patients underwent NGS using a 15-gene panel targeting autoinflammatory and immune-related genes (*ADA2, CARD14, IL10RA, LPIN2, MEFV, MVK, NLRC4, NLRP12, NLRP3, NOD2, PLCG2, PSTPIP1, SLC29A3, TMEM173, TNFRSF1A*). Patients carrying pathogenic or likely pathogenic *NOD2* variants and coexisting any other SAID-related non-pathogenic gene variants, or who met HRF or PFAPA criteria, or were followed for less than one year, were excluded.

Data collection included both retrospective and prospective components. The retrospective phase covered cases diagnosed prior to study initiation (January 1992–April 2022) with at least six months of follow-up, while the prospective phase enrolled new cases during the study period. Data on demographics, genetics, clinical features, laboratory results, imaging, histopathology, treatment, and response were systematically recorded. Treatment responses were classified as complete response (CR) for full symptom resolution with normalization of inflammatory markers, or partial response (PR) for clinical improvement requiring ongoing therapy (11).

The clinical relevance of each identified variant was assessed using multiple sources, including the Infevers Registry, ClinVar (https://www.ncbi.nlm.nih.gov/clinvar/), and Varsome (https://varsome.com/about/general/varsome-citations/), complemented by an extensive literature review. Variants were classified following the American College of Medical Genetics (ACMG) criteria as benign, likely benign, variant of uncertain significance (VUS), pathogenic, or likely pathogenic (12).

Ethical approval for this study was obtained from the Ege University Medical School Hospital Ethics Committee (approval number: 22-4T/35). All procedures adhered to the principles outlined in the Declaration of Helsinki (2013 revision) and relevant guidelines on human and animal research ethics. Written informed consent was secured from all patients and their parents prior to data collection.

For functional studies, a control group was established consisting of healthy controls (HCs) and disease controls (DCs) diagnosed with Blau syndrome (BS). The HC group included asymptomatic individuals without chronic illnesses, among whom one was a heterozygous carrier of the most frequent variant identified in the patient group, and another carried wild-type *NOD2*. The DC group consisted of BS patients harboring pathogenic *NOD2* mutations, all under care at our center.

As all patients were receiving immunomodulatory therapies, blood samples for functional assays were collected during disease flares, when markers of inflammation such as C-reactive protein (CRP) and serum amyloid A (SAA) were elevated. This allowed for the evaluation of cytokine responses and activation of downstream NF-κB and MAPK signaling pathways.

### 2.2 Functional assays

#### 2.2.1 Isolation of PBMCs and *in vitro* stimulation of cytokine production

Peripheral blood mononuclear cells (PBMCs) were isolated from whole blood using density gradient centrifugation with Lymphoprep (#07801, Stemcell Technologies). Sixteen milliliters of blood were collected into EDTA tubes and diluted at a 1:1 ratio with Dulbecco’s Phosphate-Buffered Saline (PBS) containing 2% Fetal Bovine Serum (FBS) at room temperature. The diluted samples were carefully layered over an equal volume of Lymphoprep and centrifuged at 800 × g for 20 minutes at room temperature. PBMCs were harvested from the interface, transferred to clean tubes, and washed twice with PBS by centrifugation at 300 × g. Viability and cell counts were determined using trypan blue exclusion.

PBMCs (5 × 10^6^ cells) were seeded into 25 cm² flasks and stimulated with 100 ng/ml L18-MDP, a synthetic muramyl dipeptide analog and NOD2 ligand (#tlrl-lmdp, InvivoGen) (13). Unstimulated cultures served as controls. Following 24 hours of incubation, cells and culture supernatants were harvested separately for subsequent molecular analyses.

#### 2.2.2 Cytokine Profiles

Cytokine concentrations (GM-CSF, IFN-γ, IL-2, IL-4, IL-6, IL-8, IL-10, and TNF-α) were quantified in both MDP-stimulated and control cultures using the Bio-Plex Pro Human Cytokine 8-Plex Assay (#M50000007A, Bio-Rad). The kit components included magnetic capture beads, detection antibodies, standards, and internal quality controls. Culture supernatants were obtained by centrifugation at 1,000 × g for 15 minutes at 4 °C. As the culture medium contained 10% FBS, no additional BSA stabilization was necessary. Samples were diluted 1:3 prior to analysis. The assay was carried out according to the manufacturer’s instructions, and data acquisition and cytokine quantification were performed using Bio-Plex Manager Software.

#### 2.2.3 Western blot analysis

Phosphorylation of IκBα (p-IκBα) serves as a marker of NF-κB pathway activation, as its degradation allows NF-κB to translocate to the nucleus and promote transcription of proinflammatory genes (14). Likewise, phosphorylation of p38 MAPK (p-p38) indicates activation of the MAP kinase signaling cascade (15). To evaluate the functional impact of *NOD2*variants on these pathways, levels of p-IκBα and p-p38 were measured. Proteins were extracted from PBMCs using Complete Lysis-M buffer (Roche), and concentrations were determined via the Bradford assay. Lysates (20 µg) were resolved by SDS-PAGE and transferred onto PVDF membranes. The following antibodies from Cell Signaling Technology were employed: phospho-p38 MAPK (Thr180/Tyr182, D3F9; #4511, 1:1000), phospho-IκBα (Ser32/36, 5A5; #9246, 1:1000), IκBα (#9242, 1:1000), GAPDH (#5174, 1:1000), anti-rabbit IgG (#7074, 1:2000), and anti-mouse IgG (#7076, 1:1000). Detection was performed using Clarity Western ECL Substrate (Bio-Rad), and signals were visualized with a C-DiGit Blot Scanner (LicorBio).

### 2.3 Statistical analysis

Data were analyzed using descriptive statistics, with all statistical evaluations carried out in GraphPad Prism (version 9.3). Experiments were performed in triplicate, and values are expressed as mean ± standard deviation. To assess the impact of MDP stimulation on cytokine production across different disease groups or *NOD2* variants, one-way or two-way ANOVA was employed, as appropriate. *Post hoc* multiple comparisons were performed using Tukey’s or Sidak’s test. A p-value below 0.05 was considered indicative of statistical significance.

## 3 Results

### 3.1 Demographics and clinical findings

Twelve patients (53.8% female) with *NOD2* variants classified as non-pathogenic and a diagnosis of SURF were enrolled at a median age of 14.5 years. Demographic characteristics, clinical features, and treatment data are presented in **Table 1**.

**Table 1:** The demographic features, clinical manifestations and treatments of the SURF patients.

Features	Patients (n:12,100%)
Female, n (%)	7 (58.3%)
Current age, median years (min-max)	14.5 (4–19)
Age at onset, median years (min-max)	9.25 (0.5-16)
Age at onset	
<5 years, n (%)	3 (25%)
5–10 years, n (%)	5 (41.7%)
>10 years, n (%)	4 (33.3%)
Duration follow-up, median years (min-max)	3.75 (2.5-10)
Family history positive for recurrent fever, n (%)	3 (25%)
Consanguinity, n (%)	2 (16.7%)
Chronic disease course, n (%)	4 (33.3%)
Recurrent fever	12 (100%)
Increased acute-phase reactants during disease flare	12 (100%)
Duration fever, median days (min-max)	4.5 (2–9)
Musculoskeletal involvement	8 (66.6%)
Arthritis	3 (25%)
Limb pain	5 (41.7%)
Myalgia	1 (8.3%)
Osteitis	3 (25%)
Cutaneous involvement	6 (50%)
Maculopapular rash	2 (33.3%)
Panniculitis	2 (33.3%)
Hidradenitis suppurativa	1 (16.6%)
Granulomatous dermatitis	1 (16.6%)
Pyoderma gangrenosum	1 (16.6%)
Gastrointestinal involvement	5 (41.7%)
Abdominal pain	5 (100%)
Diarrhea	2 (40%)
Vomiting	1 (20%)
Granulomatous inflammation	3 (25%)
Eye involvement	2 (16.7%)
Treatments	
Colchine, n (%)	12 (100%)
On Demand Steroids, n (%)	8 (66.6%)
DMARDs, n (%)	4 (33.3%)
Methotrexate, n (%)	2 (16.6%)
Sulfasalazine, n (%)	3 (30%)
Azatiopurine, n (%)	1 (8.3%)
Anti-TNF, n (%)	2 (16.6%)

SURF, Syndrome of Undifferentiated Recurrent Fever; TNF, Tumor Necrosis Factor.

Six distinct heterozygous *NOD2* variants were identified: R702W (n = 3), G908R (n = 2), and one case each of A1000T, L682F, P268S, L1000fs, and R753Q. Additionally, two patients carried compound heterozygous variants: P268S/R702W and P268S/V955I. Based on ACMG criteria (8), six variants were classified as variants of uncertain significance (VUS), while P268S and R753Q were deemed likely benign or benign. The healthy control group included one individual heterozygous for R702W—the most frequent variant observed in the cohort—and one with wild-type *NOD2*. The disease control group comprised two BS patients harboring the heterozygous R587C variant. Further details on *NOD2* variants in both patients and controls are provided in **Table 2**.

**Table 2:** The detailed characteristics of SURF patients and disease controls.

Variables	SURF patients												Blau syndrome	
	P1	P2	P3	P4	P5	P6	P7	P8	P9	P10	P11	P12	BS1	BS2
Age, y/Gender	6/F	5.5/M	11/F	18/M	12/F	17/F	18/F	19/M	18/F	14/F	14/M	15/M	19/M	25/F
Age at disease onset, y	1.5	2.5	8	11	1	9.5	15	16	9	9.5	9	12	8	1
Follow-up duration,y	3.5	3	2.5	7	10	4	3	3	8	4	5	3	9.5	12
NOD2 variation	R702W	G908R	L682F	L1007fs	A1000T	R753Q	P268S	P268S/V955I	G908R	R702W	R702W	R702W/ P268S	R587C	R587C
ACMG classification	VUS	VUS	VUS	VUS	VUS	LB	B	B/LB	VUS	VUS	VUS	VUS/B	LP	LP
Clinical features	Recurrent fever Abdominal pain Vomiting,	Recurrent fever, Maculo-papular rash	Recurrent fever, Limb pain	Recurrent fever, Abdominal pain, Bloody diarrhea, hidradenitis suppurativa granulomatous dermatitis	Recurrent fever, Abdominal pain, intermittent diarrhea Arthritis	Recurrent fever, Arthritis, Panniculitis	Recurrent fever, Limb pain, Unilateral granulomatous panuveitis	Recurrent fever, Bilateral granulomatous anterior uveitis	Recurrent fever, Polyarthritis Myalgia, Panniculitis	Recurrent fever, Limb pain, Osteitis Maculo-papular rash	Recurrent fever, Abdominal pain, Limb pain, Osteitis Pyoderma gangrenosum	Recurrent fever Abdominal pain Limb pain Osteitis	Recurrent fever Boggy swelling, tenosynovitis, camptodactyly, polyarthritis	Recurrent fever Boggy swelling, tenosynovitis, camptodactyly, polyarthritis bilateral granulomatous anterior uveitis Granulomatous nephritis
Complication	None	None	None	None	None	None	Ocular perforation	None	None	None	None	None	Osteoporosis	Osteoporosis, end stage renal disease
On-demand steroids	none	none	none	+ (colitis)	none	+ (Panniculitis)	+ (uveitis)	+ (uveitis)	+ (Panniculitis)	+ (osteitis)	+ (PG, osteitis)	+ (osteitis)	+	+
Colchicine	CR	CR	CR	PR	PR	PR	PR	PR	PR	PR	PR	PR	NR	NR
MTX	none	none	none	none	none	none	none	none	none	PR (osteitis)	CR (osteitis)	none	PR	PR
SSZ	none	none	none	NR	none	none	none	none	none	PR (osteitis)	none	PR (osteitis)	NR	NR
AZA	none	none	none	PR (colitis)	none	none	none	none	none	none	none	none	none	none
Anti-TNFs	none	none	none	PR (colitis)	none	none	none	none	none	CR (osteitis)	none	none	CR	CR

BS, Blau syndrome; CR, Complete treatment response; PR, Partial response; NR, Non response; P, Patient; PG, Pyoderma gangrenosum; SURF, Syndrome of Undifferentiated Recurrent Fever; y, Years; VUS, Variant of uncertain significance; LB, Likely benign; LP, Likely pathogenic; MMF, Mycophenolate mofetil; TNF, Tumor Necrosis Factor; SSZ, Sulfasalazine; MTX, Methotrexate; AZA, Azathioprine; ACMG, The American College of Medical Genetics.

Symptom onset ranged from 1 to 16 years, with the majority (n = 8; 66.6%) developing symptoms before age 10. All patients experienced recurrent fevers accompanied by elevated CRP and SAA levels during attacks. Musculoskeletal involvement occurred in two-thirds of patients, predominantly as limb pain (41.7%). Cutaneous manifestations were present in half of the cohort, presenting as maculopapular rash (n = 2), recurrent panniculitis (n = 2), pyoderma gangrenosum (n = 1), hidradenitis suppurativa (n = 1), or granulomatous dermatitis (n = 1). Gastrointestinal involvement was observed in 41.7% of patients, with abdominal pain consistently present in all cases and accompanied by diarrhea and vomiting at varying frequencies. Ocular involvement was less common, with granulomatous uveitis documented in two patients. Overall, granulomatous involvement of the skin and/or eyes was identified in 25% of the patients (n = 3) **Table 1**.

All patients were treated with colchicine, which remained part of their therapy at the last follow-up. Among them, 25% achieved complete response (CR), while 75% demonstrated partial response (PR) requiring additional treatments. Corticosteroids were administered to 8 patients (66.6%), primarily in the presence of osteitis, colitis, uveitis, or panniculitis. They were used either on demand during SURF flares with incomplete colchicine response (n = 5; 62.5%) or for flare-ups of chronic organ-specific inflammation (n = 4; 50%). All patients responded favorably to corticosteroids. Disease-modifying antirheumatic drugs (DMARDs), including methotrexate, sulfasalazine, azathioprine, and anti-TNF-α agents, were prescribed to 4 patients (33.3%) due to ongoing inflammation characterized by osteitis and colitis. Additional data on clinical features and treatment outcomes can be found in **Table 2**.

### 3.2 Comparison of cytokine profiles among HCs, DCs, and SURF patients

**Figure 1:**
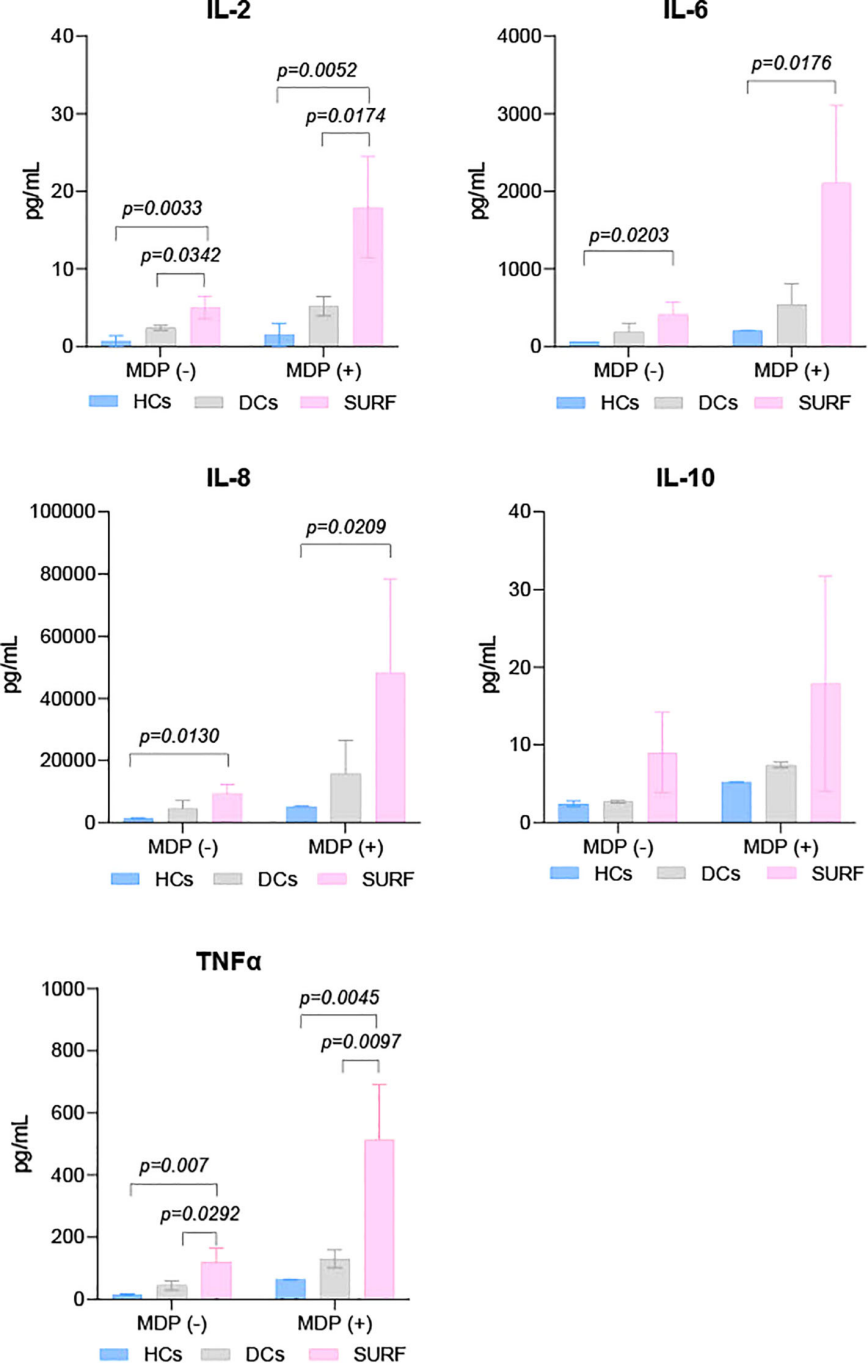
Cytokine levels in control and patient groups. HCs, Healthy controls; DCs, Disease controls, and SURF, Syndrome of Undifferentiated Recurrent Fever.

Baseline and MDP-stimulated cytokine concentrations (TNF-α, IL-2, IL-8, IFN-γ, IL-4, IL-6, IL-10, and GM-CSF) were evaluated across SURF patients, healthy controls (HCs), and disease controls (DCs). Cytokine profiles did not differ significantly between HCs and DCs under either unstimulated or MDP-stimulated conditions (**Figure 1**).

In contrast, SURF patients showed markedly elevated IL-2 and TNF-α levels compared to both HCs and DCs at baseline and following MDP exposure. IL-6 and IL-8 concentrations were also significantly higher in the SURF group versus HCs under both conditions (**Figure 1**). No statistically significant differences were identified for the other cytokines analyzed.

### 3.3 Impact of NOD2 variants on the NOD2 signaling pathway

To investigate the effect of *NOD2* variants on NF-κB and MAPK pathway activation, protein lysates were prepared from PBMCs of patients and controls at baseline and after 24 hours of MDP stimulation. Western blot analyses targeted IκBα, phosphorylated IκBα (p-IκBα), and phosphorylated p38 (p-p38, MAPK14).

**Figure 2:**
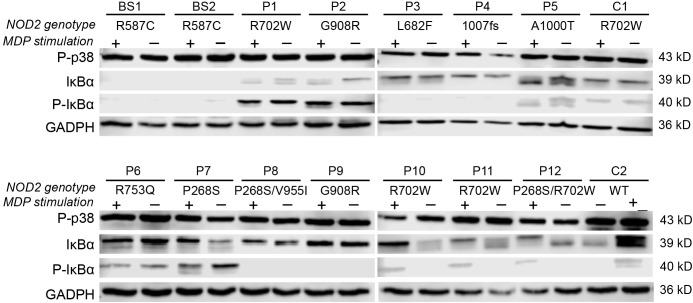
Effects of NOD2 variants on NF-κB and MAPK activation. Western blot analysis of p-p38, IκBα, and p-IκBα proteins at baseline and 24 hours post-MDP stimulation, categorized by NOD2 genotypes. GAPDH was used as a loading control.

Interestingly, p-p38 was detected across all samples—including those from healthy controls—regardless of MDP exposure (**Figure 2**). Evaluation of p-IκBα levels, combined with cytokine secretion patterns under both basal and stimulated conditions, allowed stratification of *NOD2* variants based on NF-κB pathway activity. Variants were grouped into low, moderate, or high inflammatory profiles according to NF-κB activation and associated cytokine responses.

#### 3.3.1 Impaired NF-κB activation and a low inflammatory profile

**Figure 3:**
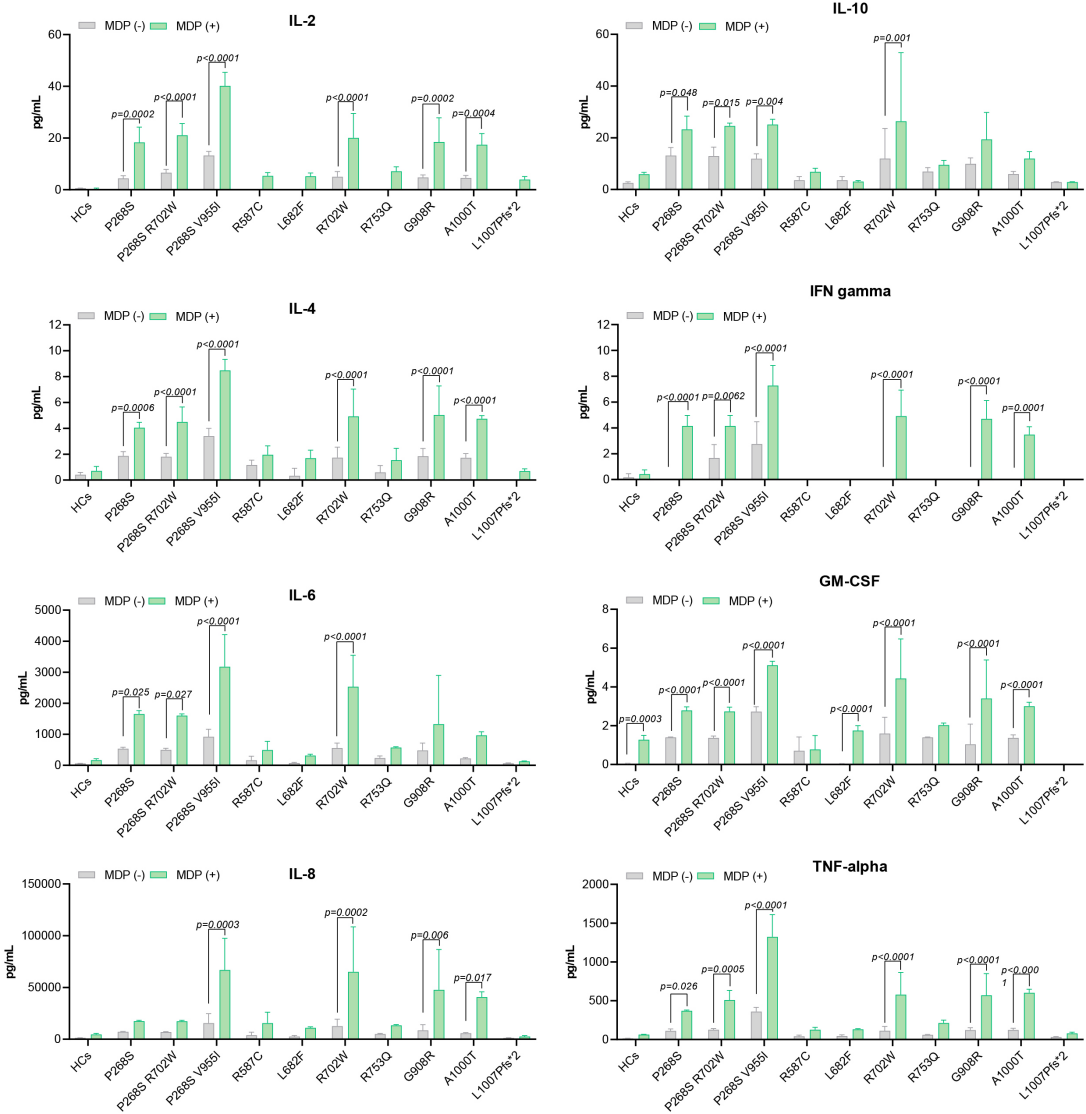
Cytokine levels stratified by NOD2 variants.

**Figure 4:**
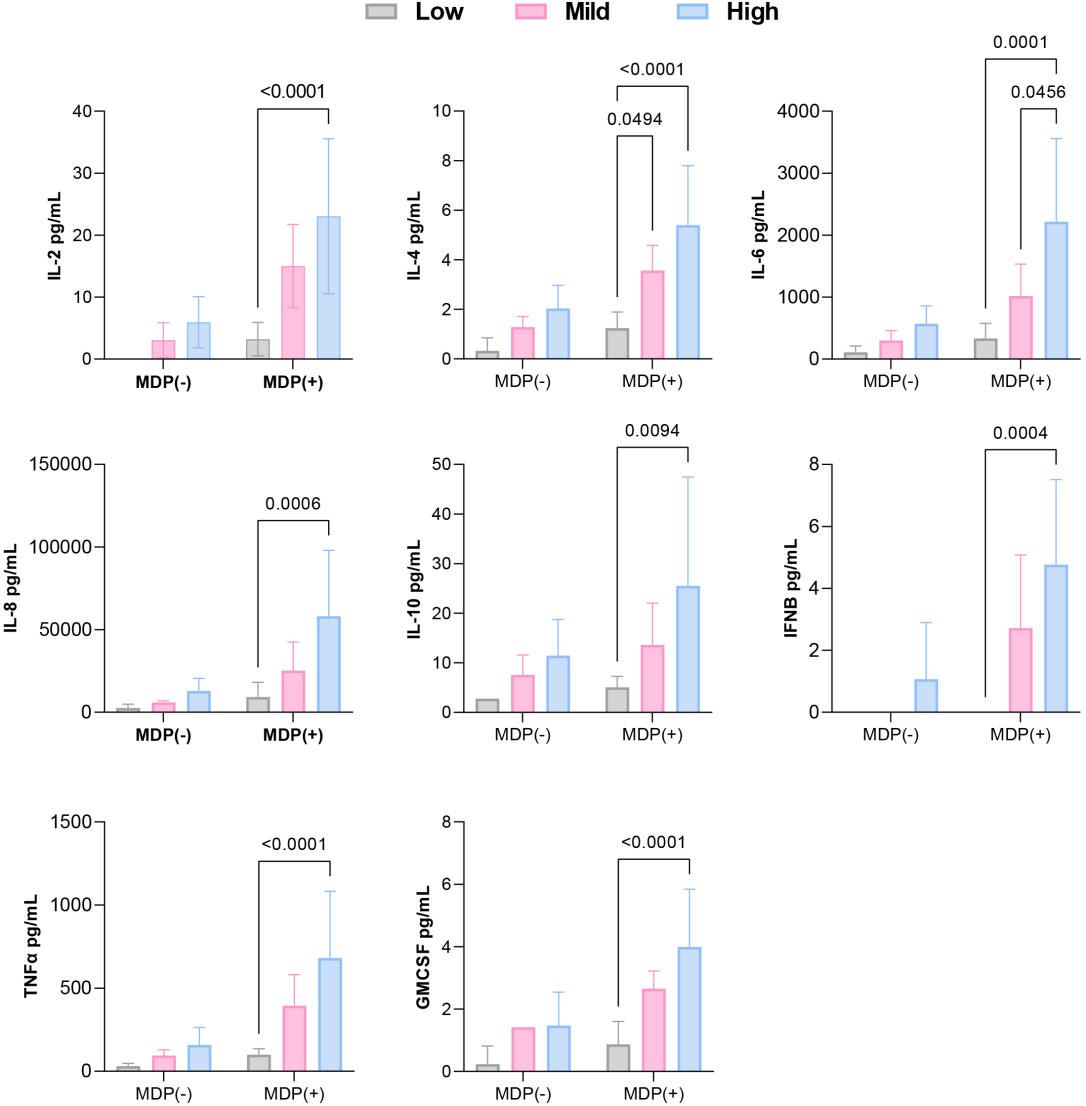
Comparison of cytokine levels among low, mild, and high inflammatory profiles, classified based on p-IκBα expression and cytokine production under baseline and post-MDP conditions.

SURF patients carrying the L682F (P3) and L1007fs (P4) variants, along with Blau syndrome cases (BS1 and BS2) harboring the R587C variant, showed absent p-IκBα expression at baseline and following MDP stimulation (**Figure 2**). Both healthy controls and the L682F carrier exhibited minimal cytokine production in response to MDP, apart from a modest increase in GM-CSF. Similarly, no statistically significant cytokine induction was observed in carriers of the R587C and L1007fs variants (**Figure 3**). Cytokine concentrations in these groups, both at rest and after stimulation, were comparable to those of healthy controls and significantly lower than levels seen in other *NOD2* variants. These findings suggest that L682F, L1007fs, and R587C are associated with a hypoinflammatory profile and impaired NF-κB pathway activation (**Figure 4**).

Notably, Patient 6 (R753Q variant) demonstrated constitutive NF-κB activation, yet did not exhibit elevated cytokine production (**Figures 3**, **4**).

#### 3.3.2 Constitutive NF-κB activation and a mild inflammatory profile

Patients with the A1000T (P5) and P268S (P7) variants displayed p-IκBα expression at both baseline and following MDP stimulation, consistent with constitutive activation of the NF-κB pathway (**Figure 2**). In the P268S carrier, MDP stimulation resulted in statistically significant elevation of all cytokines except IL-8. In contrast, the A1000T variant was associated with increased levels of all cytokines except IL-6 and IL-10 relative to healthy controls (**Figure 3**).

Although the overall cytokine production in these patients was lower than that observed in individuals carrying R702W, G908R, P268S/V955I, or R702W/P268S variants, these differences did not reach statistical significance. Collectively, these results suggest that A1000T and P268S variants promote sustained, low-grade activation of NF-κB, contributing to a moderate proinflammatory profile (**Figure 4**).

#### 3.3.3 Variable NF-κB activation and a high inflammatory profile

Patients harboring R702W (P1, P10, P11), G908R (P2, P9), P268S/V955I (P8), and R702W/P268S (P12) variants demonstrated the strongest inflammatory profiles, with MDP-stimulated cytokine production significantly higher than that of healthy controls (HCs) (**Figures 3**, **4**). Both the P268S/V955I and R702W variants were associated with elevated levels of all measured cytokines. Similarly, the P268S/R702W and G908R variants induced substantial cytokine responses, though selectively: P268S/R702W did not increase IL-8, and G908R failed to elevate IL-6 and IL-10. This pattern may point to a potent but cytokine-specific inflammatory signature.

Despite pronounced cytokine secretion, NF-κB activation patterns appeared to vary across these patients. For example, P1 (R702W) and HC1 (R702W carrier) exhibited basal p-IκBα phosphorylation, suggesting a pre-activated NF-κB state (**Figure 2**). Conversely, in P10, P11 (R702W), P12 (R702W/P268S), and HC2 (wild-type), p-IκBα was detectable only after MDP stimulation. Cytokine outputs were comparable between the two HCs; however, SURF patients with R702W variants showed significantly higher IL-6, IL-8, and TNF-α levels than HCs, both at baseline and after MDP exposure.

## 4 Discussion

Although SURF is classified as an autoinflammatory disorder without consistent associations to pathogenic gene mutations, recent studies have increasingly identified benign or VUS alterations across several genes, including NOD2, supporting the concept of a multifactorial etiology (6–9). Similarly, NOD2-associated diseases are regarded as genetically transitional diseases (GTDs), situated between monogenic and polygenic conditions, where mutations are necessary but not solely sufficient for disease onset (16). Moreover, even in monogenic Blau syndrome, reduced-penetrance variants have been documented, underscoring the complexity of NOD2-driven inflammation (17). Nevertheless, the functional impact of non-pathogenic NOD2 variants in SURF remains uncertain. Moreover, targeted NGS panels may aid diagnosis in a subset of patients with suspected AIDs, but their diagnostic yield remains limited (18), requiring clinical and functional correlation. Against this background, our study is, to the best of our knowledge, the first to investigate their functional consequences by specifically examining NF-κB activation and cytokine production. Our results demonstrate aberrant NOD2-mediated signaling, with distinct NF-κB activity and cytokine patterns that appear to be linked to specific genotypes. Notably, elevated levels of IL-2, TNF-α, IL-6, and IL-8 in SURF patients compared to HCs suggest that these cytokines may contribute to the underlying disease mechanisms.

Despite carrying the same NOD2 variant, patients exhibited variable clinical features, with recurrent fever in all cases, most frequently accompanied by abdominal and limb pain, while half of the cohort also developed cutaneous involvement in the form of various rashes. Additional manifestations such as rash, myalgia, and arthritis were in line with previous SURF cohorts (6–8, 19, 20). While some reports emphasize phenotypic variability (7, 8, 19), others suggest a more uniform presentation influenced by genetic factors (20). A chronic inflammatory course affecting the gastrointestinal tract or musculoskeletal system occurred in 33.3% of our patients—higher than the 12.3% previously reported (7)—possibly reflecting our smaller sample size or the universal presence of NOD2 variants. When compared with the limited number of published SURF cases carrying NOD2 variants (6, 8, 9), our findings were consistent in showing recurrent fever frequently accompanied by abdominal pain, musculoskeletal symptoms, and rash. Unlike the existing literature, however, rare features such as oral ulcers, pharyngitis, lymphadenopathy, hepatosplenomegaly, pericarditis, conjunctivitis, and periorbital edema were not detected. Notably, the granulomatous inflammation of the skin and eyes observed in our cohort has not been previously reported in SURF patients with NOD2 variants. Given the established link between NOD2 and granulomatous inflammation in Blau syndrome and Crohn disease (4, 5), our findings highlight the need for further studies to determine whether granulomatous inflammation constitutes a distinct phenotypic subset of SURF or merely reflects a coincidental association.

In the absence of standardized treatment protocols, SURF management typically involves colchicine, corticosteroids, and IL-21 inhibitors (21). Although prior studies have described good colchicine response rates in over half of patients (6, 7, 20), with colchicine-sensitive cases proposed to represent a more homogeneous subgroup (20), we observed fewer complete responders, possibly reflecting broader clinical variability. Recent surveys indicate that 70.8% of clinicians prescribe corticosteroids during flares based on severity rather than as routine therapy (22). Similarly, corticosteroids in our series were used on demand for disease flares or chronic organ inflammation, with all treated patients improving. Moreover, Papa et al. (6) reported that SURF patients carrying NOD2 variants demonstrated partial response to colchicine but achieved complete remission with corticosteroids, a finding consistent with our results and suggesting a potential association between NOD2 variants and limited colchicine responsiveness but favorable corticosteroid outcomes. Additionally, one-third of patients required long-term immunomodulatory therapy beyond intermittent steroids, highlighting the need for personalized treatment approaches in SURF, particularly for those with chronic or organ-specific inflammatory involvement. Larger multicenter studies will be essential to confirm these observations and refine treatment strategies in SURF.

Although cytokine patterns differ across SAIDs, with elevated cytokines often contributing to pathogenesis (23), the underlying mechanisms of SURF remain poorly defined. Moreover, current functional data are largely limited to studies comparing SURF with PFAPA or FMF (8, 11, 19). Previous studies identified a persistent IL-1 signature in SURF tonsils and noted trends toward higher IL-21β, IL-6, IL-8, and IL-17A levels in some SURF cases compared to PFAPA (8, 19). Pyrin inflammasome involvement has also been proposed in SURF pathophysiology (11). In contrast to these findings, another study demonstrated that anti–IL-1 blockade with anakinra was ineffective in three out of four colchicine-resistant patients, suggesting an IL-1β–independent inflammatory pattern in this subgroup (6). In our cohort, we observed significantly elevated IL-2 levels, a cytokine primarily implicated in Treg homeostasis in autoimmune conditions (24). In FMF, Treg dysfunction has been proposed; for instance, Rimar et al. (25) described Treg expansion following attacks, while another study noted elevated IL-10 and TGF-β, both known Treg inducers (26). However, contrasting data indicate reduced Treg numbers in FMF, suggesting impaired regulatory capacity may exacerbate disease severity (27). Elevated sIL-2R levels in FMF patients, even during remission, correlated with erythrocyte sedimentation rate and activated CD4+CD69+ T cells, implicating IL-2 signaling in persistent low-grade inflammation (28). These findings raise the possibility that IL-2-driven immune activation in SURF may reflect altered Treg function, contributing to immune dysregulation.

Variants like L1007fs, G908R, and R702W are associated with both CD and NAID. While functional studies have classified these variants as loss-of-function in CD due to reduced NF-κB activation (29–31), IBD models and CD patients without *NOD2* mutations also display impaired proinflammatory cytokine production (32, 33). This diminished response appears independent of *NOD2* genotype and distinct from healthy controls (34), suggesting additional contributors to defective immunity in CD.

Conversely, G908R has been linked to enhanced IL-8 and TNF-α production in familial sarcoidosis, despite reduced NF-κB signaling, pointing to macrophage-driven chronic inflammation (35). Although some NAID-associated *NOD2* variants act as gain-of-function mutations, compound heterozygous IVS8 + 158 and R702W variants have shown loss-of-function features in NAID, yet R702W may have different effects in NAID versus CD, consistent with distinct clinical presentations (36, 37). Our findings suggest that R702W, G908R, P268S/V955I, and R702W/P268S may be associated with robust proinflammatory cytokine responses despite varied NF-κB activation, differing from their reported loss-of-function classification in CD (29, 30). This may indicate that *NOD2* variants act differently depending on disease context, possibly through compensatory signaling or interactions with other innate immune pathways (38). Tissue-specific *NOD2* expression and local immune environments may also shape these responses.

Notably, while the R702W variant was present in both healthy control and SURF patients, only the latter displayed markedly elevated cytokine levels, indicating this variant may contribute to inflammation under disease conditions but not in healthy states. Furthermore, patients carrying P268S/V955I (P8) and G908R (P9) showed high cytokine production at baseline and after MDP stimulation, even in the absence of detectable NF-κB activation, implying alternative inflammatory pathways may be involved. Interestingly, G908R appeared to exhibit divergent NF-κB activation between two patients: one (P2) had baseline and MDP-induced p-IκBα expression, while the other (P9) lacked p-IκBα under both conditions, highlighting the influence of additional regulatory mechanisms on NF-κB signaling. Despite these differences, both patients produced significantly elevated cytokine levels compared to HCs.

Another important finding was that the L682F and L1007fs variants in SURF patients, along with R587C in BS, were suggestive of an association with a hypoinflammatory profile, characterized by absent NF-κB activation and diminished cytokine responses compared to HCs and other *NOD2* variants. The SURF patient carrying L1007fsinsC (P4) presented with chronic gastrointestinal symptoms (nonspecific focal colitis) but lacked endoscopic, radiologic, or histologic evidence of IBD, consistent with the expected loss-of-function phenotype seen in CD (29, 30). Although R587C is classified as pathogenic in BS, its functional role remains debated (39–42). Matsuda et al. (40) reported weak spontaneous NF-κB activation with R587C compared to R334W, while our data—aligned with Parkhouse et al. (41)— seem to support a hypoinflammatory effect, reinforcing emerging evidence of loss-of-function features in BS-related *NOD2* variants (42).

The R753Q variant showed cytokine levels comparable to HCs both at baseline and after MDP stimulation, despite constitutive NF-κB activation, which may suggest minimal impact on *NOD2*-driven immune responses and seems to support its classification as likely benign. In contrast, A1000T and P268S variants appeared to be associated with NF-κB activation and mild inflammatory activity—greater than HCs but lower than more strongly proinflammatory variants. Although P268S has not been shown to significantly alter *NOD2* function (29, 30), its potential role in autoinflammation remains uncertain. Given the limited functional data on A1000T and P268S, further investigation is needed to clarify their contributions in SURF.

Detection of p-p38 across all samples, including HCs and regardless of MDP stimulation, suggests that p38 MAPK activation may occur independently of *NOD2*-mediated signaling. Possible explanations may include activation via alternative pathways or basal cellular stress in PBMCs, with handling and culture conditions potentially contributing (43). This may underscores the complexity of p38 MAPK regulation and the need for further study into its broader role in immune modulation and inflammation in SURF.

Our study’s primary limitation was the small cohort, attributable to the rarity of SURF and funding constraints that limited control recruitment. The lack of whole exome sequencing (WES) and segregation analyses may have led to undetected rare pathogenic variants in other genes. Nonetheless, it should be noted that many patients with undefined autoinflammatory syndromes remain without a genetic diagnosis even after comprehensive genomic testing (44). Furthermore, while samples were obtained during disease flares, studying treatment-naïve patients in larger cohorts will be essential to better elucidate disease mechanisms. Despite these challenges, a major strength of our work is the integration of long-term clinical follow-up with functional analysis of *NOD2* variants, which may provide valuable insights into potential disease pathways.

Overall, our findings suggest that *NOD2* variants may act as disease modulators rather than primary drivers of SURF. This may highlight the importance of further functional studies to evaluate *NOD2* signaling within disease-relevant immune contexts, as identical mutations might trigger inflammation through distinct molecular routes depending on the condition. Future research should explore additional genetic, epigenetic, or environmental contributors that may influence inflammatory responses in SURF, helping to refine our understanding of *NOD2*-linked autoinflammatory disorders.

## Data Availability

The original contributions presented in the study are included in this article/tables and figures. Further inquiries can be directed to the corresponding author.
